# Eating Behavior, Food Consumption Frequency, and Nutritional Status Among Undergraduate Nursing Students: A Cross‐Sectional Study

**DOI:** 10.1002/fsn3.72376

**Published:** 2026-09-17

**Authors:** Maria Laura Lacerda Nascimento, Aline Nataly Soares Vital, Taisy Cinthia Ferro Cavalcante, Emily Pereira de Souza, Amanda Alves Marcelino da Silva

**Affiliations:** ^1^ Nursing Academic Board, Petrolina Campus University of Pernambuco Petrolina PE Brazil; ^2^ Graduate Program in Rehabilitation and Functional Performance, Petrolina Campus University of Pernambuco Petrolina PE Brazil; ^3^ Nutrition Academic Board, Petrolina Campus University of Pernambuco Petrolina PE Brazil

**Keywords:** eating behavior, food frequency questionnaire, nursing students, nutritional status

## Abstract

University nursing students face academic and lifestyle pressures that may compromise food choices and nutritional status. This study evaluated the relationship between eating behavior, food consumption frequency, and nutritional status in this population. Cross‐sectional study with 61 undergraduate nursing students. BMI was calculated from measured weight and height (WHO criteria). Food consumption frequency was assessed using the FFQ‐ELSA‐Brasil (76 items) and eating behavior using the TFEQ‐21 (Cognitive Restraint, Uncontrolled Eating, Emotional Eating). Spearman's correlation and Kruskal–Wallis test were applied (*α* = 0.05). The sample was predominantly female (93.4%), young (93.4% aged 18–25 years), and eutrophic (65.0%; mean BMI: 23.07 ± 4.19 kg/m^2^), with 15.0% overweight, 11.7% underweight, and 8.3% Grade I obesity. The TFEQ‐21 assessment showed a median Cognitive Restraint score of 50.00 (IQR = 45.45–54.55), a median Uncontrolled Eating score of 59.26 (IQR = 51.85–66.67), and a median Emotional Eating score of 55.56 (IQR = 50.00–72.22). Food consumption was based on rice, beans, and bread; fruit and vegetable diversity was limited; coffee was consumed daily or weekly by 72.1%. No significant associations were found between BMI and any TFEQ‐21 domain (all *p* > 0.05). Nursing students showed a traditional Brazilian dietary profile with limited food diversity and frequent coffee consumption. No association between BMI and eating behavior domains was detected in this sample; given the small, unadjusted convenience sample, potentially meaningful associations cannot be excluded. Health promotion strategies addressing dietary diversity and eating behavior are recommended.

## Graphical abstract

In a cross‐sectional study of 61 undergraduate nursing students in Brazil, dietary patterns were centered on rice, beans, and bread, with limited fruit and vegetable diversity and frequent coffee consumption (72.1% daily or weekly). Despite a predominantly eutrophic BMI profile (65.0%), eating behavior (TFEQ‐21 domains: Cognitive Restraint, Uncontrolled Eating, Emotional Eating) showed no significant association with BMI. These findings highlight the need for health promotion strategies targeting dietary diversity independent of anthropometric status.

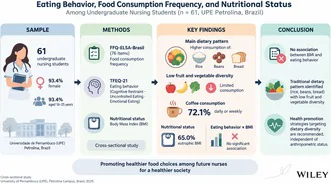

## 1 Introduction

In recent decades, Brazil has undergone changes in eating behavior associated with economic, demographic, and cultural shifts, a process known as nutritional transition. This set of changes is related to the growing search for practical, easy‐to‐prepare foods, which are generally industrialized products that are poor in nutrients and have high caloric density, gradually replacing fresh foods (Feitosa et al. 2010).

In this context, an individual's eating behavior is characterized by a set of cognitions and feelings that are reflected in their food choices. Thus, food goes beyond biological needs and becomes an expression of identity, associated with pleasure, family, culture, and interpersonal relationships. Considering the aspects mentioned regarding the determinants of such behavior, psychosocial factors are fundamental to understanding the complex relationship between food, the individual, and the social environment in which they are embedded (Cambraia 2004).

Within this framework, when analyzing the behavior of the population in general, it is evident that university students constitute a relevant group that is more susceptible to fragile eating behaviors (Costa et al. 2017). These patterns are characterized by low nutritional quality, irregular meals, and frequent consumption of ultra‐processed foods, in addition to behavioral factors such as emotional eating, cognitive restraint, and loss of control over eating (Natacci and Ferreira Júnior 2011a). Such a pattern represents an important metabolic risk factor, as it favors inadequate intake of essential nutrients and excessive consumption of simple sugars, saturated fats, and sodium, triggering the activation of inflammatory, hormonal, and metabolic mechanisms (Louzada et al. 2015). These mechanisms contribute to the development of insulin resistance, dyslipidemia, and weight gain, which are associated with the development of non‐communicable chronic diseases (NCDs) (Malta et al. 2011).

Entering university involves changes for many young people related to health care and weight gain, as they are in a period of adaptation to a new reality (Dahl et al. 2025). In this regard, university students do not always have family support for food acquisition and preparation. Moreover, their eating behavior is strongly influenced by factors such as lack of time to prepare complete meals due to high academic demands, which also interferes with food choices and favors the replacement of main meals with quick, practical, and high‐calorie snacks (Feitosa et al. 2010).

Given this scenario, the present research is justified by the need to understand the relationship between eating behavior and nutritional status, considering the importance of adequate and healthy nutrition for individuals' quality of life, which may lead to the proposal of solutions and guide care practices within the identified context. Therefore, the aim of this study was to evaluate the relationship between eating behavior, food consumption frequency, and the nutritional status of nursing students, considering possible associations between eating habits, dietary choices, and health indicators.

## 2 Materials and Methods

### 2.1 Participants

This is a cross‐sectional field study with a quantitative approach, aimed at identifying the association between eating behavior and nutritional status among Nursing students. The research was conducted at the Petrolina campus of the University of Pernambuco, located on BR‐203 Highway, km 2, 721 km from the state capital, Recife, Brazil. The study population consisted of undergraduate students aged between 18 and 45 years enrolled in a Nursing program at the Petrolina campus of the University of Pernambuco.

**FIGURE 1:**
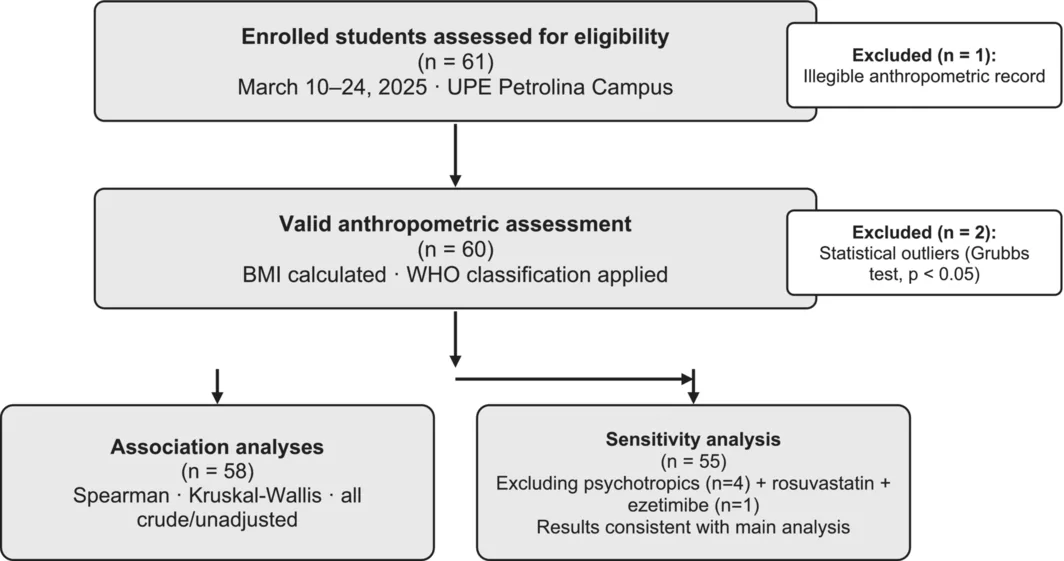
Participant flow and analytic denominators. UPE = University of Pernambuco, BMI = Body Mass Index. Grubbs test applied to BMI values.

Students aged between 18 and 45 years who were regularly enrolled in the Nursing program at the University of Pernambuco, Petrolina campus, and who agreed to participate in the study by signing the Informed Consent Form were included (Figure 1). Students older than 45 years (excluded to minimize the influence of age‐related metabolic and hormonal changes on nutritional status and eating behavior) were excluded. Regarding medication use, the operational exclusion criterion was defined as the current use of medications with a recognized direct pharmacological effect on lipid or carbohydrate metabolism (e.g., fibrates, thiazolidinediones, insulin sensitizers). Participants using other medications—including oral contraceptives (*n* = 3), psychotropic agents (*n* = 4), isotretinoin (*n* = 2), rosuvastatin + ezetimibe (*n* = 1), salbutamol (*n* = 1), and losartan (*n* = 1)—were retained in the main analysis, as these were not considered to meet the pre‐specified exclusion criterion. It is acknowledged, however, that some of these agents (particularly psychotropic medications and rosuvastatin + ezetimibe) may plausibly influence appetite, body weight, or eating behavior; this is addressed in the sensitivity analysis described below. The sample was selected by convenience, totaling 61 students, and data collection took place between March 10 and March 24, 2025.

A sensitivity analysis was conducted to assess the potential influence of medications plausibly related to appetite, body weight, or eating behavior on the main findings. Participants using psychotropic agents (*n* = 4) and rosuvastatin + ezetimibe (*n* = 1)—totalling five participants—were excluded, yielding a restricted analytic sample of *n* = 55 for the sensitivity analysis. Spearman's correlation coefficients between BMI and TFEQ‐21 domain scores were recalculated in this restricted sample. Results were consistent with the main analysis: no statistically significant associations were observed for Cognitive Restraint (*ρ* = +0.128; *p* = 0.361), Uncontrolled Eating (*ρ* = +0.039; *p* = 0.781), or Emotional Eating (*ρ* = −0.024; *p* = 0.864), indicating that the inclusion of these participants did not materially alter the conclusions of the study.

### 2.2 Instrumentation

To conduct the study, anthropometric data collection and interpretation, eating behavior, and dietary intake were used as techniques. The instruments included a portable digital electronic scale (Omron HBF 514C) and a stadiometer to assess body mass index (BMI); a semi‐structured sociodemographic questionnaire developed by the authors; a Food Frequency Questionnaire (FFQ—reduced ELSA‐BRASIL version), validated by Mannato (2013); and the Three‐Factor Eating Questionnaire‐21 (TFEQ‐21) in its version translated and validated into Portuguese by Natacci and Ferreira Júnior (2011b).

### 2.3 Procedure

The study followed the guidelines and standards regulating research involving human subjects, as set forth in Resolution No. 466/2012 (issued by the National Health Council of Brazil). It was duly submitted to and approved by the Research Ethics Committee of the University of Pernambuco (CEP‐UPE) and the Ethics Committee of the Amaury de Medeiros Integrated Health Center (CISAM), under approval Opinion 6.732.232 dated March 22, 2024.

Participants were evaluated individually at the Nervous System and Metabolism Research Laboratory, University of Pernambuco, on previously scheduled dates and times, in a reserved environment to ensure privacy and minimize external interference. Before any procedures, all participants signed the Informed Consent Form, formally agreeing to participate in the study. Prior to the assessment, standardized instructions were provided: participants were advised not to consume alcoholic beverages within 48 h before the evaluation, to maintain a 12‐h fast (including water), to avoid intense physical exercise within the preceding 24 h, to wear light clothing, and to empty their bladder before the beginning of the procedures. These precautions aimed to reduce potential physiological biases and ensure greater reliability and accuracy of the results. Data collection was carried out individually at previously scheduled times to avoid interference with academic activities. Initially, anthropometric measurements were obtained for body mass index (BMI) calculation. Body weight was measured using a portable digital electronic scale (Omron HBF 514C), with participants standing still in the center of the scale, wearing light clothing and no shoes. Height was measured using a portable stadiometer, with participants standing upright, feet together, heels against the wall, and the head positioned in the Frankfurt horizontal plane. BMI was calculated as weight (kg) divided by height squared (m^2^). Subsequently, participants completed the instruments in the following order: the sociodemographic questionnaire, the Food Frequency Questionnaire (FFQ—reduced ELSA‐Brasil version), and the Three‐Factor Eating Questionnaire‐21 (TFEQ‐21), used to assess eating behavior. The original FFQ of ELSA‐Brasil contained 114 food items and was specially created to evaluate habitual diet. Using multiple linear regression, taking into account both the frequency of consumption and the nutritional composition of the foods, the original list was reduced to 76 food items, which explained 70% of the energy variability, while maintaining relatively good capacity to measure energy and selected nutrients. The questionnaire asks participants to report how often they consumed each food item across predefined frequency categories—ranging from daily consumption to never or almost never—allowing the classification of dietary patterns by food group. Eating behavior was assessed using the Three‐Factor Eating Questionnaire in its 21‐item revised version (TFEQ‐R21). The TFEQ‐R21 is a scale that measures three domains of eating behavior: cognitive restraint (CR), uncontrolled eating (UE), and emotional eating (EE). Cognitive restraint refers to the conscious restriction of food intake aimed at controlling body weight or promoting weight loss; uncontrolled eating refers to the tendency to eat more than usual due to a loss of control over intake associated with a subjective feeling of hunger; and emotional eating refers to the inability to control eating in response to negative emotional states such as anxiety, sadness, or stress. The instrument is self‐administered and uses a 4‐point response format for items 1 to 20, and an 8‐point numerical rating scale for item 21. The TFEQ is recognized as one of the most widely used instruments for studying eating behavior, having been validated across multiple languages and cultural contexts.

### 2.4 Data Analysis

Data were initially described using descriptive statistics (Reis 2008). Categorical variables were presented as absolute and relative frequencies (%). The normality of quantitative variables was assessed using the Shapiro–Wilk test. Variables with normal distribution were described as mean and standard deviation, while those with asymmetric distribution were presented as median and interquartile range. The association between body mass index (BMI) and the domain scores of the Three‐Factor Eating Questionnaire‐21 (TFEQ‐21) was evaluated using Spearman's correlation coefficient. Differences in TFEQ‐21 domain scores among BMI categories were analyzed using the Kruskal–Wallis test. A significance level of 5% (*p* < 0.05) was adopted. Data were organized in Microsoft Excel 2016 spreadsheets and analyzed using Biostat 2009 Professional 5.8.4 for Windows.

## 3 Results

Table 1 presents the sociodemographic data of the 61 students evaluated. The majority were female (93.4%), aged between 18 and 25 years (93.4%), living with their parents or guardians (67.2%), and, for the most part, purchasing and preparing their own food (50.8%). It is noteworthy that 6.6% of the sample (*n* = 4) reported current use of psychotropic medications at the time of data collection, despite not reporting a formal diagnosis of a mental disorder (Table 1). This discrepancy likely reflects underdiagnosis, stigma‐related underreporting, or off‐label prescribing, and is acknowledged as a methodological limitation. Given that psychotropic agents may influence appetite, body weight, and eating behavior, a sensitivity analysis excluding these participants (and one additional participant using rosuvastatin + ezetimibe, a lipid‐lowering agent) was performed and is described in the Methods section. Additionally, one participant reported use of rosuvastatin + ezetimibe; although this combination has a recognized effect on lipid metabolism, this participant was retained in the main analysis as the original exclusion criterion was operationally defined prior to data collection and is addressed in the sensitivity analysis.

**TABLE 1:** Sociodemographic characteristics and lifestyle of nursing students enrolled in a health sciences program at the University of Pernambuco, Petrolina Campus, 2025 (*n* = 61).

Variables (*n* = 61)	*n*	%
Sex		
Female	57	93.4
Male	4	6.6
Age (years)		
≤ 25	57	93.4
26–35	4	6.6
Marital status		
Single	60	98.4
Married	1	1.6
Area of residence		
Urban	58	95.1
Rural	3	4.9
Academic semester		
2nd	26	42.6
4th	32	52.5
8th	3	4.9
Family income		
< 1 minimum wage	12	19.7
1–3 minimum wages	36	59.0
> 3 minimum wages	13	21.3
Lives with parents or guardians		
Yes	41	67.2
No	20	32.8
Responsible for food purchase/preparation		
Yes	31	50.8
No	30	49.2
Smoking		
Yes	3	4.9
No	58	95.1
Alcohol consumption		
Yes	24	39.3
No	37	60.7
Medication use		
Yes	12	19.7
No	49	80.3
Medications currently in use^a^		
Oral contraceptives	3	4.9
Psychotropic agents	4	6.6
Isotretinoin	2	3.3
Rosuvastatin + ezetimibe	1	1.6
Salbutamol	1	1.6
Losartan	1	1.6
Reported health conditions^b^		
None	53	86.9
Anemia	1	1.6
Arterial hypertension	1	1.6
Asthma	5	8.2
Endometriosis	1	1.6

^a^ Participants may use more than one medication concurrently; percentages calculated relative to total sample (*n* = 61).

^b^ Some participants reported more than one health condition; percentages calculated relative to total sample (*n* = 61). Minimum wage refers to the official Brazilian minimum monthly salary (BRL 1621.00 ≈ USD 295) at the time of data collection.

The assessment of the students' nutritional status analyzed variables such as height, weight, and BMI (Table 2). The classification obtained from this evaluation showed that 65.0% of the students were eutrophic, 15.0% were overweight, 11.7% were underweight, and 8.3% had Grade I obesity. BMI cut‐off values used were: underweight (< 18.5 kg/m^2^), eutrophic (18.5–24.9 kg/m^2^), overweight (25.0–29.9 kg/m^2^), and Grade I obesity (30.0–34.9 kg/m^2^), as defined by the World Health Organization (WHO).

**TABLE 2:** Anthropometric characteristics of nursing students at the University of Pernambuco, Petrolina Campus, 2025.

Continuous variables (*n* = 60^a^)					
Variable	*n*	Mean ± SD	Median	Min–Max	95% CI of mean
Age (years)	60	20.83 ± 2.55	20.00	18.00–31.00	20.19–21.48
Height (m)	60	1.65 ± 0.08	1.65	1.40–1.86	1.63–1.67
Weight (kg)	60	63.16 ± 14.06	60.65	42.30–116.00	59.61–66.72
BMI (kg/m^2^)	60	23.07 ± 4.19	22.48	16.04–33.53	22.00–24.13

BMI classification—WHO (2000)					
Classification	*n*	%	Mean BMI ± SD	BMI range (WHO)	95% CI of mean BMI
Underweight	7	11.7%	17.31 ± 0.91	< 18.5 kg/m^2^	16.47–18.15
Eutrophic	39	65.0%	21.81 ± 1.78	18.5–24.9 kg/m^2^	21.23–22.39
Overweight	9	15.0%	28.25 ± 0.67	25.0–29.9 kg/m^2^	27.73–28.77
Obesity Grade I	5	8.3%	31.60 ± 1.41	30.0–34.9 kg/m^2^	29.85–33.35

*Note:* BMI classification according to WHO (2000): Underweight < 18.5; Eutrophic 18.5–24.9; Overweight 25.0–29.9; Obesity Grade I 30.0–34.9 kg/m^2^.

Abbreviations: BMI = Body Mass Index, CI = confidence interval, Max = maximum, Min = minimum, SD = standard deviation.

^a^ One participant was excluded due to illegible anthropometric record.

Eating behavior was assessed in all 61 participants using the TFEQ‐21 (Table 3). Normality was evaluated by the Shapiro–Wilk test: Cognitive Restraint (CR; W = 0.969, *p* = 0.139) and Uncontrolled Eating (UE; W = 0.981, *p* = 0.485) followed normal distributions, while Emotional Eating (EE; W = 0.957, *p* = 0.039) did not. Median and IQR are the primary descriptive measures for EE; means ± SD are provided for all domains for comparison. CR: median 50.00 (IQR: 45.45–54.55; mean ± SD: 50.97 ± 9.25; 95% CI: 48.65–53.29). UE: median 59.26 (IQR: 51.85–66.67; mean ± SD: 58.04 ± 11.25; 95% CI: 55.22–60.87). EE: median 55.56 (IQR: 50.00–72.22; mean ± SD: 56.92 ± 22.03; 95% CI: 51.39–62.45), with the widest dispersion, indicating marked individual variation in emotional eating (Table 3).

**TABLE 3:** Eating behavior domain scores among nursing students—TFEQ‐21 (*n* = 61).

Domain (items)	Median (IQR)	Mean ± SD	Min–Max	95% CI of mean	Shapiro–Wilk (*p*)
Cognitive restraint—CR (Q1, Q5, Q11, Q17, Q18, Q21)	50.00 (45.45–54.55)	50.97 ± 9.25	27.27–72.73	48.65–53.29	0.1392
Uncontrolled eating—UE (Q3, Q6, Q8, Q9, Q12, Q13, Q15, Q19, Q20)	59.26 (51.85–66.67)	58.04 ± 11.25	33.33–85.19	55.22–60.87	0.4847
Emotional eating—EE (Q2, Q4, Q7, Q10, Q14, Q16)	55.56 (50.00–72.22)	56.92 ± 22.03	5.56–100.00	51.39–62.45	0.0394

*Note:* Scores normalized to 0–100 scale. Higher scores indicate greater expression of each domain. Normality assessed by Shapiro–Wilk test (*α* = 0.05). CR and UE follow normal distribution (parametric); EE does not (*p* = 0.039; non‐parametric). Median (IQR) is the primary descriptive measure for EE; Mean ± SD is reported additionally for all domains. *Source:* Natacci and Ferreira Júnior (2011b).

Abbreviations: CR = cognitive restraint; EE = emotional eating; IQR = interquartile range (Q25–Q75); UE = uncontrolled eating.

The detailed food frequency data are presented in Supporting Information: Table (*n* = 61). The dietary pattern was predominantly structured around rice (daily or weekly: 100.0%), beans (88.5%), French bread (90.2%), and farofa/couscous (88.5%). Beef and chicken breast were consumed weekly by 73.8% and 68.9%, with daily intake by a further 11.5% and 14.8%, respectively. Eggs: daily or weekly by 96.7%. Fish intake was low: 52.5% never/rarely consumed boiled fish; 45.9% fried fish. Banana was the most frequent fruit (daily or weekly: 80.3%); papaya (62.3% never/rarely), pineapple (42.6%), and watermelon (39.3%) were infrequent. Onion (95.1%) and garlic (91.8%) were nearly universally consumed as condiments. Low vegetable diversity: 63.9% rarely/never consumed kale/spinach, 80.3% cauliflower, 65.6% broccoli. Sweets mostly occasional: chocolate weekly (37.7%) or never/rarely (24.6%); ice cream monthly (44.3%) or never/rarely (34.4%); pizza monthly (52.5%) or never/rarely (26.2%). Coffee: 44.3% daily, 27.9% weekly. Natural juice: 75.4% daily or weekly. Soft drinks: 49.2% weekly, 27.9% never/rarely. Industrialized juice (60.7% never/rarely) and artificial juice (70.5% never/rarely) were uncommon. Alcoholic beverages predominantly not consumed: beer (82.0% never/rarely), wine (72.1%), spirits (82.0%).

Among protein sources, options such as beef, chicken, and eggs showed an intermediate pattern, generally consumed 2–4 times per week or weekly. Fish consumption was low, with most participants reporting rare or no intake. Dairy products (cheese and yogurt) showed an irregular pattern, predominantly with occasional frequencies (1–3 times per month or never).

Regarding fruits, bananas were the most frequently consumed, with daily or almost daily intake reported by a significant portion of the sample. Other fruits, such as grapes, apples/pears, and mangoes, appeared in intermediate patterns, being consumed weekly. Pineapple and orange/tangerine showed low frequency, being mainly reported as monthly or never consumed. Among vegetables, lettuce, tomato, and carrot were consumed weekly (2–4 times per week). However, vegetables such as okra, green beans, cauliflower, and broccoli were mostly reported as never or almost never consumed, revealing low variety intake within this group.

With respect to sweets and ultra‐processed foods, occasional or absent consumption predominated. Chocolate and ice cream, for example, were consumed sporadically, with more than half of participants reporting a frequency of 1–3 times per month or never. Pudding, plain cake, and cookies followed the same pattern.

Beverage consumption showed a diverse pattern. Soft drinks were consumed occasionally, between 2 and 4 times per week or monthly, but with a substantial group reporting no consumption. Artificial and industrialized juices were predominantly reported as never or almost never consumed, while natural juices had greater presence, especially with weekly consumption. Alcoholic beverages, such as beer, wine, and spirits, were predominantly reported as never consumed.

**FIGURE 2:**
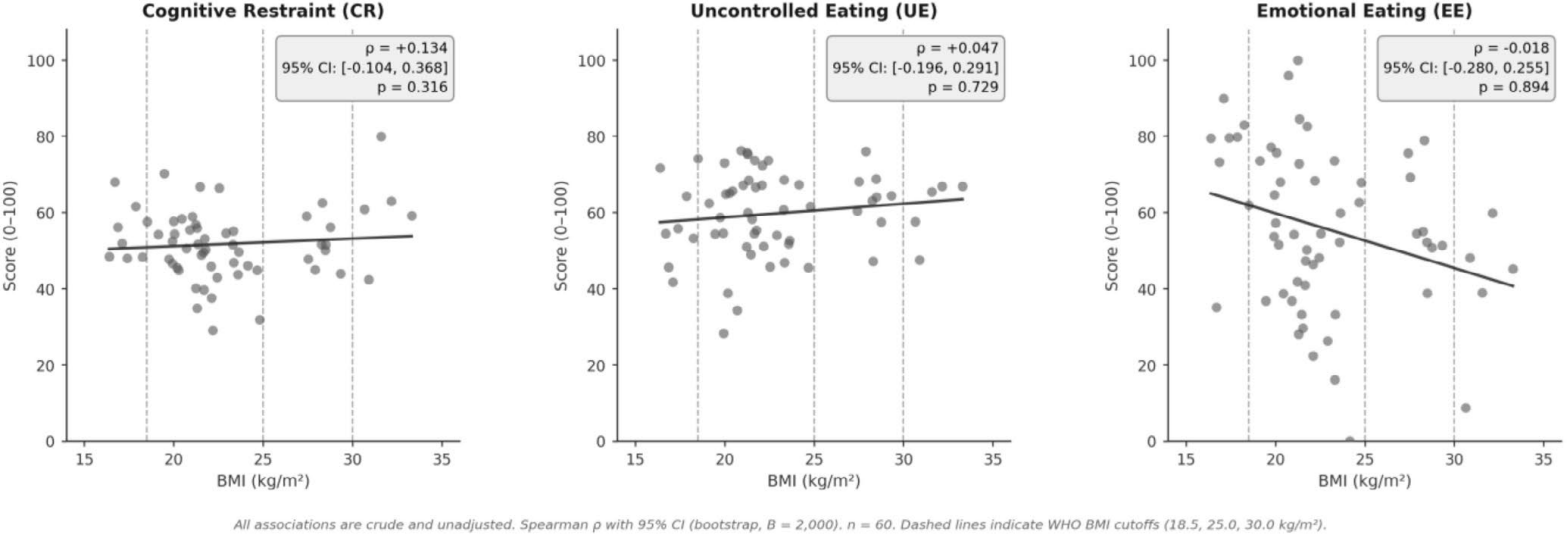
Scatterplots of BMI versus TFEQ‐21 eating behavior domain scores (*n* = 60, UPE Petrolina, 2025). Each panel shows individual data points, a linear trend line, and Spearman *ρ* with 95% CI (bootstrap, *B* = 2000). Dashed lines: WHO BMI cutoffs. All associations crude and unadjusted. CR = cognitive restraint, UE = uncontrolled eating, EE = Emotional eating.

**FIGURE 3:**
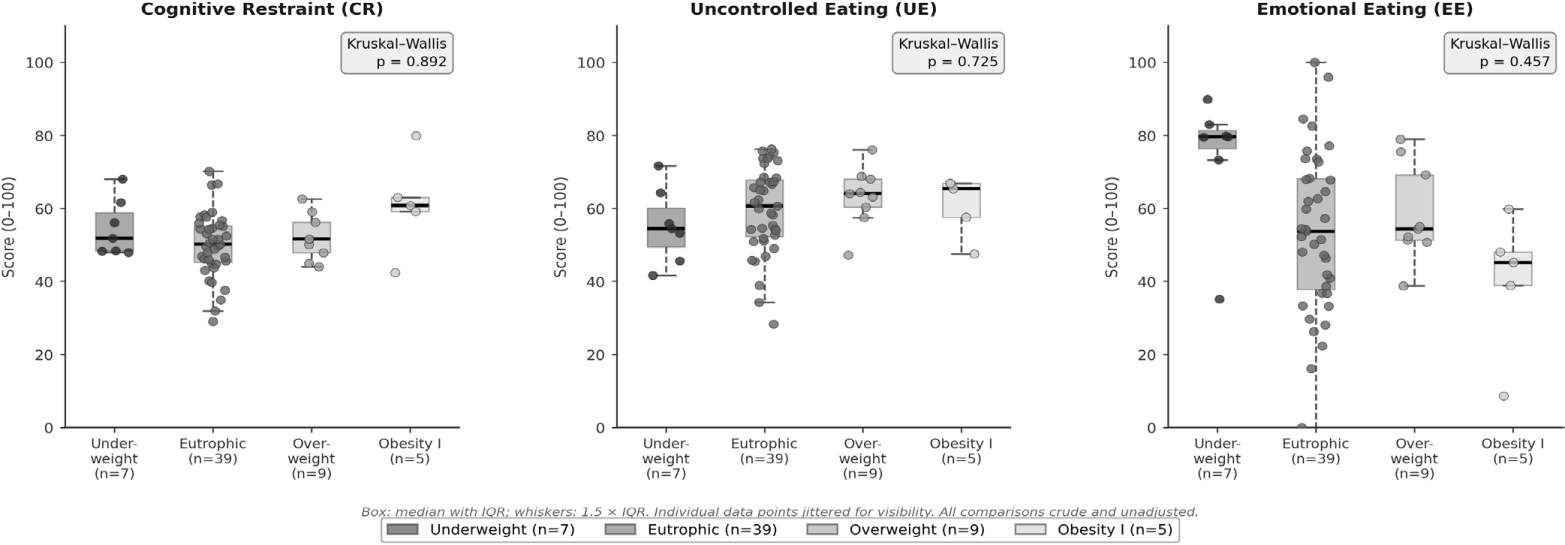
TFEQ‐21 domain scores (Cognitive Restraint, Uncontrolled Eating, Emotional Eating) by BMI category, displayed as jittered dot and box plots for the full anthropometric sample (*n* = 60; Underweight *n* = 7, Eutrophic *n* = 39, Overweight *n* = 9, Obesity Grade I *n* = 5), consistent with Table 2. A sensitivity analysis excluding two participants identified as statistical outliers (Grubbs test, *p* < 0.05) yields *n* = 58; consequently, the Kruskal–Wallis *p*‐values from that sensitivity analysis (cognitive restraint *p* = 0.892; uncontrolled eating *p* = 0.725; emotional eating *p* = 0.457) differ slightly from the primary‐analysis figures reported in Table 4, though both analyses converge on the same conclusion of no statistically significant differences in eating‐behavior domains across BMI categories.

Statistical analyses were conducted on the complete valid anthropometric sample (*n* = 60). A sensitivity analysis excluding two participants identified as statistical outliers (Grubbs test, *p* < 0.05) is reported in Table 4 (*n* = 58). All associations are crude and unadjusted. No statistically significant correlations were observed between BMI and any TFEQ‐21 domain: Cognitive Restraint (*ρ* = +0.134; 95% CI: −0.104 to +0.368; *p* = 0.316), Uncontrolled Eating (*ρ* = +0.047; 95% CI: −0.196 to +0.291; *p* = 0.729), or Emotional Eating (*ρ* = −0.018; 95% CI: −0.280 to +0.255; *p* = 0.894) (Table 4; Figures 2 and 3).

**TABLE 4:** Spearman correlation coefficients (*ρ*) between BMI and TFEQ‐21 eating behavior domains (*n* = 60 primary; *n* = 58 sensitivity^a^).

Domain	*ρ* (Spearman)	95% CI (*ρ*)^b^	*p*
Cognitive restraint (CR)	+0.134	−0.104; +0.368	0.316
Uncontrolled eating (UE)	+0.047	−0.196; +0.291	0.729
Emotional eating (EE)	−0.018	−0.280; +0.255	0.894

Domain	Underweight (*n* = 6) Md (IQR)	Eutrophic (*n* = 38) Md (IQR)	Overweight (*n* = 9) Md (IQR)	Obesity I (*n* = 5) Md (IQR)	*H* *p*‐value Sig.
Cognitive restraint (CR)	50.0 (46.6–50.0)	52.3 (45.5–58.0)	50.0 (45.5–54.5)	50.0 (50.0–54.5)	*H* = 0.817 *p* = 0.845 ns
Uncontrolled eating (UE)	59.3 (56.5–59.3)	59.3 (49.1–66.7)	55.6 (44.4–59.3)	55.6 (51.9–63.0)	*H* = 1.627 *p* = 0.653 ns
Emotional eating (EE)	69.4 (62.5–80.6)	50.0 (45.8–72.2)	50.0 (50.0–55.6)	66.7 (66.7–77.8)	*H* = 3.598 *p* = 0.308 ns

*Note:* Kruskal–Wallis Test: TFEQ‐21 Domain Scores by BMI Category (*n* = 60 primary; *n* = 58 sensitivity).

Abbreviations: *ρ* = Spearman correlation coefficient, IQR = interquartile range, Md = median, ns = not significant (*p* ≥ 0.05).

^a^ Primary analysis: *n* = 60 (all valid anthropometric records). Sensitivity analysis excludes two participants identified as statistical outliers (Grubbs test, *p* < 0.05), yielding *n* = 58; results were consistent with the primary analysis. All associations reported are crude (unadjusted); the small BMI subgroup sizes (Underweight *n* = 7; Obesity I *n* = 5) limit statistical power. Non‐significant *p*‐values should not be interpreted as evidence of no association.

^b^ 95% CI obtained by bootstrap (*B* = 2000).

The Kruskal–Wallis test likewise revealed no statistically significant differences in TFEQ‐21 domain scores across BMI categories: Cognitive Restraint (*H* = 0.817; *p* = 0.845), Uncontrolled Eating (*H* = 1.627; *p* = 0.653), and Emotional Eating (*H* = 3.598; *p* = 0.308) (Table 4). Given the small subgroup sizes—Underweight (*n* = 6) and Grade I Obesity (*n* = 5)—these analyses have limited statistical power, and non‐significant *p*‐values should not be interpreted as evidence of absence of association.

The sample showed a predominance of eutrophic individuals. Regarding eating behavior, no significant correlations were observed between BMI and any of the domains evaluated by the TFEQ‐21. In addition, the comparison of domain scores across BMI categories also revealed no statistically significant differences. These results indicate that no statistically significant differences were detected in this small sample.

## 4 Discussion

The present study reveals a predominance of female participants (93.4%). This proportion is consistent with the feminized profile of Nursing courses in Brazil: according to the 2023 Higher Education Census conducted by the Instituto Nacional de Estudos e Pesquisas Educacionais Anísio Teixeira (INEP), approximately 73.8% of graduates in the Health Sciences area are female, with Nursing programs historically presenting even higher proportions (INEP 2023). The predominance observed in this sample therefore reflects the actual enrollment profile of the course rather than a sampling bias. Nevertheless, this composition limits the generalizability of the findings to male students, and results should be interpreted within this demographic context. Current data regarding the distribution of women in higher education also reflect broader changes in social paradigms and the implementation of public policies characterized by the increasing presence of women in academic contexts (INEP 2023).

Based on the results obtained, it is observed that most of the sample is responsible for purchasing and preparing their own food, which may represent a risk, considering that the participants are enrolled in a full‐time academic program. This scenario reflects reduced time availability due to academic activities, favoring the replacement of complete meals with quick, high‐calorie‐density snacks (Feitosa et al. 2010). In this sense, a decline in dietary quality is a long‐term risk factor for chronic diseases, in addition to contributing to unfavorable weight gain and changes in body composition that interfere with quality of life (Olatona et al. 2018).

In this study, a predominance of eutrophy among participants was observed; however, the percentages of overweight and underweight raise an important warning regarding the monitoring of the nutritional status of this sample. It should be emphasized that BMI analysis, although not sufficient on its own to assess cardiometabolic risk, represents a relevant indicator in the initial identification of nutritional alterations. Overweight, as estimated by BMI, is directly related to the risk of early development of several non‐communicable chronic diseases (NCDs), such as Type II diabetes and cardiovascular diseases, as it represents a marker of metabolic imbalance which, when developed in youth, tends to progress over the years (World Health Organization 2025). Thus, even with a majority of eutrophic individuals, the presence of overweight cases highlights the need for preventive health interventions aimed at promoting healthy habits within the university context.

The dietary pattern [The detailed dietary consumption data are presented in the Supporting Information] identified among participants reveals a strong base composed of rice, beans, and bread, characterizing the main structure of the diet. This combination reflects the traditional Brazilian dietary profile, especially in urban regions, which accounted for 95.1% of the studied sample. The daily consumption of these foods reinforces the sociocultural role of eating, in which food goes beyond biological needs and becomes an expression of identity associated with pleasure, family, culture, and interpersonal relationships (Cambraia 2004).

Animal protein sources showed moderate frequency, with emphasis on beef and chicken. These foods play a central role in the Brazilian diet, being important sources of protein as well as micronutrients such as iron, zinc, and B‐complex vitamins. The predominance of these foods may be associated with their relatively greater economic accessibility compared to other protein sources and their versatility in preparation (Brazil, Ministry of Health 2014).

Fruit and vegetable consumption was restricted to a few more common items, while other vegetables showed low intake frequency. The low and limited variety of fruit and vegetable intake indicates compromised nutritional diversity and may directly limit the supply of essential nutrients, vitamins, and minerals necessary for proper body functioning. When associated with the observed sociodemographic and academic profile, this finding reinforces the nutritional vulnerability of this group.

These findings are broadly consistent with the dietary quality deficits reported by Llanaj et al. (2021) in a large cross‐sectional study among Hungarian adults (*n* = 703), in which poor adherence to established healthy dietary patterns—including the Healthy Diet Indicator (HDI), the Dietary Approaches to Stop Hypertension (DASH) score, and the nutrient‐based EAT‐Lancet index—was observed independently of ethnic background, sex, or socioeconomic status. Despite the substantial methodological differences between that study (nutrient‐based dietary indexes derived from 24‐h recall) and the present work (food consumption frequency assessed by FFQ in a convenience sample of nursing students), the convergence of findings is noteworthy: in both contexts, limited fruit and vegetable diversity, low adherence to health‐promoting dietary recommendations, and the need for institutional nutrition interventions emerged as central concerns. Llanaj et al. further highlighted that poor dietary quality was not an isolated phenomenon but reflected systemic deficiencies in nutrition education and preventive services—a conclusion that resonates with the present study's observation that nursing students, despite their health sciences training, exhibited dietary patterns that deviated from recommended diversity targets (Llanaj et al. 2021).

Sweet foods and ultra‐processed products appeared occasionally, and sugary and alcoholic beverages showed low frequency, although they were not absent. Coffee, in turn, showed notable prevalence, with nearly half of participants reporting daily consumption. Frequent coffee consumption among university students is frequently reported in national and international studies, generally associated with the lifestyle of this life stage (Ferreira and Queiroz 2020). Thus, although moderate caffeine intake offers benefits such as reduced fatigue and increased concentration, excessive consumption may lead to adverse effects such as insomnia, anxiety, and dependence. In the study population, high intake may be related to high demands and pressures for performance and productivity (Fischer et al. 2021).

Although the results of the present study did not identify significant associations between BMI and eating behavior factors (cognitive restraint, uncontrolled eating, and emotional eating), evidence from international literature points to different results. In a study conducted in Poland, for example, cognitive restraint showed a positive correlation with BMI in men (*r* = 0.174; *p* = 0.036) and women (*r* = 0.239; *p* < 0.001), while emotional eating was positively associated with BMI only in women (*r* = 0.184; *p* = 0.008), suggesting that individuals with higher BMI tend to adopt more restrictive or emotional eating behaviors (Gębski et al. 2021). Similarly, a multicenter study conducted among university students in the Iberian Peninsula found that emotional eating was more frequent among women and was associated with obesity, corroborating patterns observed across different cultural and academic contexts (Sosa‐Cordobés et al. 2024). It should be noted, however, that the methodological approaches differ substantially: while the present study used Spearman's correlation and Kruskal–Wallis tests on crude, unadjusted associations in a small convenience sample (*n* = 60), studies reporting significant BMI–eating behavior associations typically employ larger samples with adjusted regression models accounting for relevant confounders such as sex, age, and socioeconomic status—an analytical approach not feasible in the present dataset. In this regard, the work of Llanaj et al. (2021) underscores the importance of multivariable adjustment when examining diet–health relationships in cross‐sectional designs, as unadjusted estimates may obscure associations that become apparent only after controlling for key covariates.

In a broader context, an investigation conducted among adults identified significant associations between emotional eating—characterized by eating in response to emotions such as stress, anxiety, or fatigue—and external eating, defined as the consumption of meals and foods prepared outside the home, with BMI, especially among individuals with obesity, while cognitive restraint did not show a consistent relationship (Gębski et al. 2021). Furthermore, other studies indicate that psychosocial factors, such as perceived stress, may intensify disordered eating behaviors, including emotional eating and uncontrolled eating, regardless of BMI category—an aspect that is particularly relevant in the university context (Oliveira and Hutz 2010).

Taken together, the findings of this study have important implications for school health policy, practice, and equity, particularly in higher education institutions that train future health professionals. Although most students were eutrophic, the presence of overweight and obesity in 23% of the sample, along with low dietary diversity and frequent coffee consumption, highlights the need for structured institutional strategies. Universities should promote healthier food environments aligned with national dietary guidelines, expand access to affordable fruits and vegetables, and implement routine nutritional screening programs integrated with mental health support, recognizing that eating behaviors are influenced by psychosocial factors beyond BMI.

From a practical perspective, no association between BMI and eating behavior domains was detected in this sample; given the small, unadjusted convenience sample, potentially meaningful associations cannot be excluded. Screening tools such as the TFEQ‐21 may complement nutritional assessments, while health promotion programs should address time management for meal preparation, emotional eating, excessive caffeine intake, and improvement of dietary diversity through interdisciplinary approaches.

Equity considerations are also central, as many students are responsible for purchasing and preparing their own food while managing full‐time academic demands, which may function as structural barriers to healthy eating. Institutional actions such as subsidized healthy meals, food assistance initiatives, flexible academic policies, and culturally sensitive nutrition education are essential. University students represent a transitional group in which lifelong habits are consolidated, and therefore institutions have both an ethical and educational responsibility to foster environments that integrate nutrition, mental health, and equity into campus policy and practice.

## 5 Conclusion

The present study showed that university students, mostly women, exhibit eating patterns influenced by academic, social, and cultural factors, which directly affect food choices and nutritional status. Despite the predominance of eutrophy, the percentages of overweight and obesity account for 23% of the evaluated population, highlighting the presence of these outcomes and reinforcing the need for attention to conditions that may favor the development of non‐communicable chronic diseases (NCDs).

In this context, although sweets, ultra‐processed foods, and alcoholic beverages appeared only occasionally, frequent coffee consumption stands out, associated with the academic lifestyle and the demands of this life stage, which deserves attention to prevent adverse effects of this practice.

As limitations of the study, the recruitment of the sample stands out, since the intense academic routine of the students made it difficult to coordinate schedules for questionnaire administration and the collection of anthropometric data. This reality directly influenced the sample size, representing an important methodological challenge. Even so, the findings contribute to understanding eating behavior among university students and reinforce the importance of health promotion plans that consider the multiple dimensions of eating behavior within this context. A further limitation concerns the clinical characterization of participants: the presence of psychotropic medication use (6.6%) in the absence of a reported mental disorder diagnosis introduces uncertainty regarding the completeness of clinical data. Although a sensitivity analysis excluding participants on medications plausibly related to appetite, weight, or eating behavior yielded results consistent with the main findings, the small subgroup sizes preclude definitive conclusions, and this should be considered when interpreting the reported associations.

## Author Contributions

**Maria Laura Lacerda Nascimento:** investigation, writing – original draft, methodology. **Aline Nataly Soares Vital:** methodology. **Taisy Cinthia Ferro Cavalcante:** formal analysis, writing – review and editing. **Emily Pereira de Souza:** conceptualization, methodology, writing – review and editing, formal analysis, supervision. **Amanda Alves Marcelino da Silva:** conceptualization, investigation, funding acquisition, writing – review and editing, methodology, validation, formal analysis, project administration, supervision.

## Funding

Institutional Scientific Initiation Program of the University of Pernambuco, PFA (SEI Process No. 0040608324.000071/2024‐20).

## Ethics Statement

This study was approved by the Research Ethics Committee of the University of Pernambuco (CEP‐UPE) and the Ethics Committee of the Amaury de Medeiros Integrated Health Center (CISAM), under approval Opinion 6,732,232, dated March 22, 2024. The study was conducted in accordance with Resolution No. 466/2012 of the National Health Council of Brazil.

## Consent

Informed consent was obtained from all participants prior to data collection.

## Conflicts of Interest

The authors declare no conflicts of interest.

## Supporting information


**Data S1:** fsn372376‐sup‐0001‐Supinfo1.docx.

**Data S2:** fsn372376‐sup‐0002‐Supinfo2.docx.

## Data Availability

The data supporting the findings of this study are available from the corresponding author upon reasonable request.
