# Supplementary material for: Eating Behavior, Food Consumption Frequency, and Nutritional Status Among Undergraduate Nursing Students: A Cross‐Sectional Study
Source: Food Sci Nutr. 2026 Sep 17;14(9):e72376. doi: 10.1002/fsn3.72376 (PMC13586782; doi:10.1002/fsn3.72376)
Supplement: Supplementary file 1 [file FSN3-14-e72376-s001.docx]

| **Supplementary Table – Frequency of Food Consumption by Food Group Nursing Students — University of Pernambuco, Petrolina Campus, 2025 (n = 61)** | | | | | | |
| --- | --- | --- | --- | --- | --- | --- |
|  | | | | | | |
| **No.** | **Food Item** | **Daily ≥1x/day n (%)** | **Weekly 2–6x/week n (%)** | **Monthly 1–3x/month n (%)** | **Never/Almost Never n (%)** | **Seasonal n (%)** |
| **CEREALS, BREADS AND TUBERS** | | | | | | |
| **1** | **Rice** | **28 (45.9%)** | **33 (54.1%)** | **0 (0.0%)** | **0 (0.0%)** | **—** |
| 2 | Oats/Granola/Cereals | 7 (11.5%) | 18 (29.5%) | 5 (8.2%) | 31 (50.8%) | — |
| **3** | **Farofa/Couscous** | **12 (19.7%)** | **42 (68.9%)** | **4 (6.6%)** | **3 (4.9%)** | **—** |
| 4 | Cassava/Corn Flour | 3 (4.9%) | 22 (36.1%) | 6 (9.8%) | 30 (49.2%) | — |
| 5 | Light Bread | 7 (11.5%) | 21 (34.4%) | 2 (3.3%) | 31 (50.8%) | — |
| **6** | **French Bread** | **17 (27.9%)** | **38 (62.3%)** | **2 (3.3%)** | **4 (6.6%)** | **—** |
| 7 | Sliced/Homemade Bread | 1 (1.6%) | 15 (24.6%) | 10 (16.4%) | 35 (57.4%) | — |
| 8 | Whole Wheat Bread | 9 (14.8%) | 14 (23.0%) | 2 (3.3%) | 36 (59.0%) | — |
| 9 | Cheese Bread | 1 (1.6%) | 9 (14.8%) | 19 (31.1%) | 32 (52.5%) | — |
| 10 | Plain Cake | 0 (0.0%) | 23 (37.7%) | 27 (44.3%) | 11 (18.0%) | — |
| 11 | Savory Crackers | 3 (4.9%) | 12 (19.7%) | 14 (23.0%) | 32 (52.5%) | — |
| 12 | Sweet Cookies | 4 (6.6%) | 20 (32.8%) | 8 (13.1%) | 29 (47.5%) | — |
| 13 | Polenta/Cornmeal Porridge | 0 (0.0%) | 3 (4.9%) | 6 (9.8%) | 50 (82.0%) | 2 (3.3%) |
| 14 | Potato | 4 (6.6%) | 30 (49.2%) | 17 (27.9%) | 10 (16.4%) | — |
| 15 | Cassava/Plantain/Sweet Potato | 3 (4.9%) | 32 (52.5%) | 14 (23.0%) | 12 (19.7%) | — |
|  |  |  |  |  |  |  |
| **FRUITS** | | | | | | |
| 16 | Orange/Tangerine | 3 (4.9%) | 21 (34.4%) | 15 (24.6%) | 22 (36.1%) | — |
| **17** | **Banana** | **12 (19.7%)** | **37 (60.7%)** | **7 (11.5%)** | **5 (8.2%)** | **—** |
| 18 | Papaya | 0 (0.0%) | 17 (27.9%) | 6 (9.8%) | 38 (62.3%) | — |
| 19 | Apple/Pear | 4 (6.6%) | 18 (29.5%) | 20 (32.8%) | 17 (27.9%) | 2 (3.3%) |
| 20 | Watermelon | 1 (1.6%) | 12 (19.7%) | 24 (39.3%) | 24 (39.3%) | — |
| 21 | Melon | 3 (4.9%) | 17 (27.9%) | 15 (24.6%) | 26 (42.6%) | — |
| 22 | Pineapple | 1 (1.6%) | 10 (16.4%) | 24 (39.3%) | 26 (42.6%) | — |
| 23 | Mango | 2 (3.3%) | 22 (36.1%) | 22 (36.1%) | 15 (24.6%) | — |
| 24 | Grape | 4 (6.6%) | 18 (29.5%) | 23 (37.7%) | 16 (26.2%) | — |
|  |  |  |  |  |  |  |
| **VEGETABLES AND GREENS** | | | | | | |
| 25 | Lettuce | 2 (3.3%) | 34 (55.7%) | 12 (19.7%) | 13 (21.3%) | — |
| 26 | Kale/Spinach | 3 (4.9%) | 8 (13.1%) | 11 (18.0%) | 39 (63.9%) | — |
| 27 | Cabbage | 2 (3.3%) | 16 (26.2%) | 9 (14.8%) | 34 (55.7%) | — |
| 28 | Chicory/Watercress/Arugula | 0 (0.0%) | 3 (4.9%) | 7 (11.5%) | 51 (83.6%) | — |
| 29 | Tomato | 7 (11.5%) | 33 (54.1%) | 7 (11.5%) | 14 (23.0%) | — |
| 30 | Pumpkin | 2 (3.3%) | 14 (23.0%) | 13 (21.3%) | 32 (52.5%) | — |
| 31 | Zucchini/Chayote | 1 (1.6%) | 11 (18.0%) | 9 (14.8%) | 40 (65.6%) | — |
| 32 | Green Beans | 0 (0.0%) | 1 (1.6%) | 2 (3.3%) | 58 (95.1%) | — |
| 33 | Okra | 0 (0.0%) | 5 (8.2%) | 8 (13.1%) | 47 (77.0%) | 1 (1.6%) |
| **34** | **Onion** | **28 (45.9%)** | **30 (49.2%)** | **2 (3.3%)** | **1 (1.6%)** | **—** |
| **35** | **Garlic** | **26 (42.6%)** | **30 (49.2%)** | **3 (4.9%)** | **2 (3.3%)** | **—** |
| **36** | **Carrot** | **6 (9.8%)** | **37 (60.7%)** | **9 (14.8%)** | **8 (13.1%)** | **1 (1.6%)** |
| 37 | Beet | 2 (3.3%) | 19 (31.1%) | 15 (24.6%) | 24 (39.3%) | 1 (1.6%) |
| 38 | Cauliflower | 0 (0.0%) | 6 (9.8%) | 6 (9.8%) | 49 (80.3%) | — |
| 39 | Broccoli | 2 (3.3%) | 4 (6.6%) | 14 (23.0%) | 40 (65.6%) | 1 (1.6%) |
| 40 | Sweet Corn | 2 (3.3%) | 10 (16.4%) | 17 (27.9%) | 30 (49.2%) | 2 (3.3%) |
|  |  |  |  |  |  |  |
| **LEGUMES AND OILSEEDS** | | | | | | |
| **41** | **Black/Kidney Beans** | **19 (31.1%)** | **35 (57.4%)** | **3 (4.9%)** | **4 (6.6%)** | **—** |
| 42 | Lentil/Chickpea/Pea | 1 (1.6%) | 2 (3.3%) | 6 (9.8%) | 51 (83.6%) | 1 (1.6%) |
| 43 | Walnuts/Chestnuts/Almonds/Pistachios | 2 (3.3%) | 5 (8.2%) | 20 (32.8%) | 34 (55.7%) | — |
|  |  |  |  |  |  |  |
| **EGGS AND DAIRY PRODUCTS** | | | | | | |
| **44** | **Egg** | **21 (34.4%)** | **38 (62.3%)** | **1 (1.6%)** | **1 (1.6%)** | **—** |
| **45** | **Milk** | **10 (16.4%)** | **41 (67.2%)** | **6 (9.8%)** | **4 (6.6%)** | **—** |
| 46 | Yogurt | 1 (1.6%) | 27 (44.3%) | 17 (27.9%) | 15 (24.6%) | 1 (1.6%) |
| 47 | White Cheeses | 8 (13.1%) | 31 (50.8%) | 7 (11.5%) | 15 (24.6%) | — |
| 48 | Yellow Cheeses | 6 (9.8%) | 35 (57.4%) | 5 (8.2%) | 15 (24.6%) | — |
| **49** | **Margarine/Vegetable Cream** | **18 (29.5%)** | **31 (50.8%)** | **2 (3.3%)** | **10 (16.4%)** | **—** |
|  |  |  |  |  |  |  |
| **MEATS AND PROCESSED MEATS** | | | | | | |
| 50 | Tripe/Offal | 0 (0.0%) | 3 (4.9%) | 5 (8.2%) | 53 (86.9%) | — |
| **51** | **Beef** | **7 (11.5%)** | **45 (73.8%)** | **5 (8.2%)** | **4 (6.6%)** | **—** |
| 52 | Pork | 0 (0.0%) | 21 (34.4%) | 12 (19.7%) | 27 (44.3%) | 1 (1.6%) |
| **53** | **Chicken Breast** | **9 (14.8%)** | **42 (68.9%)** | **5 (8.2%)** | **5 (8.2%)** | **—** |
| 54 | Cooked Chicken | 6 (9.8%) | 36 (59.0%) | 8 (13.1%) | 11 (18.0%) | — |
| 55 | Sausage | 2 (3.3%) | 17 (27.9%) | 16 (26.2%) | 26 (42.6%) | — |
| 56 | Ham/Mortadella/Salami | 3 (4.9%) | 25 (41.0%) | 12 (19.7%) | 21 (34.4%) | — |
| 57 | Cooked Fish | 2 (3.3%) | 5 (8.2%) | 18 (29.5%) | 32 (52.5%) | 4 (6.6%) |
| 58 | Fried Fish | 2 (3.3%) | 4 (6.6%) | 24 (39.3%) | 28 (45.9%) | 3 (4.9%) |
|  |  |  |  |  |  |  |
| **PREPARATIONS AND ULTRA-PROCESSED FOODS** | | | | | | |
| 59 | Pizza | 0 (0.0%) | 12 (19.7%) | 32 (52.5%) | 16 (26.2%) | 1 (1.6%) |
| 60 | Pasta | 3 (4.9%) | 38 (62.3%) | 15 (24.6%) | 5 (8.2%) | — |
| 61 | Baked Savory Pastries | 2 (3.3%) | 25 (41.0%) | 17 (27.9%) | 17 (27.9%) | — |
| 62 | Acarajé (Bean Fritter) | 0 (0.0%) | 3 (4.9%) | 13 (21.3%) | 44 (72.1%) | 1 (1.6%) |
| 63 | Stroganoff | 0 (0.0%) | 18 (29.5%) | 26 (42.6%) | 16 (26.2%) | 1 (1.6%) |
| 64 | Vegetable Soup | 1 (1.6%) | 10 (16.4%) | 17 (27.9%) | 33 (54.1%) | — |
|  |  |  |  |  |  |  |
| **SWEETS AND DESSERTS** | | | | | | |
| 65 | Creamy Ice Cream | 2 (3.3%) | 11 (18.0%) | 27 (44.3%) | 21 (34.4%) | — |
| 66 | Chocolate | 3 (4.9%) | 23 (37.7%) | 20 (32.8%) | 15 (24.6%) | — |
| 67 | Pudding/Milk-Based Sweets/Mousse | 2 (3.3%) | 8 (13.1%) | 18 (29.5%) | 31 (50.8%) | 2 (3.3%) |
|  |  |  |  |  |  |  |
| **BEVERAGES** | | | | | | |
| 68 | Soft Drink | 4 (6.6%) | 30 (49.2%) | 10 (16.4%) | 17 (27.9%) | — |
| **69** | **Coffee** | **27 (44.3%)** | **17 (27.9%)** | **2 (3.3%)** | **15 (24.6%)** | **—** |
| **70** | **Natural Juice** | **10 (16.4%)** | **36 (59.0%)** | **13 (21.3%)** | **2 (3.3%)** | **—** |
| 71 | Industrialized Juice | 1 (1.6%) | 9 (14.8%) | 14 (23.0%) | 37 (60.7%) | — |
| 72 | Artificial Juice | 0 (0.0%) | 5 (8.2%) | 13 (21.3%) | 43 (70.5%) | — |
| 73 | Chimarrão (Mate Tea) | 0 (0.0%) | 0 (0.0%) | 0 (0.0%) | 61 (100.0%) | — |
| 74 | Beer | 0 (0.0%) | 1 (1.6%) | 9 (14.8%) | 50 (82.0%) | 1 (1.6%) |
| 75 | Wine | 0 (0.0%) | 2 (3.3%) | 14 (23.0%) | 44 (72.1%) | 1 (1.6%) |
| 76 | Distilled Alcoholic Beverages | 0 (0.0%) | 1 (1.6%) | 9 (14.8%) | 50 (82.0%) | 1 (1.6%) |
|  |  |  |  |  |  |  |
|  |  |  |  |  |  |  |
| **★ Highlighted foods = frequent consumption (daily + weekly) ≥ 70% of participants.** | | | | | | |
| *Daily ≥1x/day n (%)* | | | | | | |
