# Supplementary material for: Eating Behavior, Food Consumption Frequency, and Nutritional Status Among Undergraduate Nursing Students: A Cross‐Sectional Study
Source: Food Sci Nutr. 2026 Sep 17;14(9):e72376. doi: 10.1002/fsn3.72376 (PMC13586782; doi:10.1002/fsn3.72376)
Supplement: Supplementary file 2 [file FSN3-14-e72376-s002.docx]

| **Missing Data Statement — Questionnaire-Level Missingness** | | | | | | | |
| --- | --- | --- | --- | --- | --- | --- | --- |
| *Nursing Students — UPE Petrolina, 2025 \| Enrolled n = 61 (anthropometry n = 60; 1 record excluded due to illegible data)* | | | | | | | |
| **Instrument / Section** | **Items (n)** | **Enrolled (n)** | **Complete (n)** | **Missing (n)** | **Missingness (%)** | **Handling rule** | **Basis** |
| **SOCIODEMOGRAPHIC QUESTIONNAIRE** | | | | | | | |
| Sociodemographic questionnaire | 15 | 61 | 61 | 0 | 0,0% | Complete-case; no imputation | Self-report; no item-level gaps observed |
| **ANTHROPOMETRIC ASSESSMENT** | | | | | | | |
| Height & weight (BMI) | 2 | 61 | 60 | 1 | 1,6% | 1 record excluded (illegible); analysis n=60 | Measured; excluded before analysis |
| **FOOD FREQUENCY QUESTIONNAIRE (QFA-ELSA-Brasil, 76 items)** | | | | | | | |
| Any individual food item | 76 | 61 | 61 | 0 | 0,0% | Missing item = "never/almost never" (cat. 1) | Standard protocol (Mannato et al., 2013) |
| **THREE-FACTOR EATING QUESTIONNAIRE (TFEQ-R21)** | | | | | | | |
| Items 1–20 (4-point Likert) | 20 | 61 | 61 | 0 | 0,0% | Complete-case; no imputation | All items completed by all participants |
| Item 21 (8-point scale) | 1 | 61 | 61 | 0 | 0,0% | Complete-case; no imputation | All items completed by all participants |
| Domain scores (CR, UE, EE) | 3 | 61 | 61 | 0 | 0,0% | Computed for all 61 participants | No partial scores required; n=61 for TFEQ analyses |
|  |  |  |  |  |  |  |  |
| *Notes: Gray cells = no missing data (complete case). One anthropometric record was excluded due to illegibility (n=60 for BMI analyses). All questionnaire data (QFA and TFEQ-R21) were complete for all 61 enrolled participants. Multiple imputation was considered unnecessary given absence of item-level missingness. CR = Cognitive Restraint; UE = Uncontrolled Eating; EE = Emotional Eating.* | | | | | | | |
